# An expanded reference catalog of translated open reading frames for biomedical research

**DOI:** 10.1093/nar/gkag234

**Published:** 2026-03-24

**Authors:** Sonia Chothani, Jorge Ruiz-Orera, Jack A S Tierney, Michal I Swirski, Hakon Tjeldnes, Leron W Kok, Jim Clauwaert, Eric W Deutsch, M Mar Alba, Julie L Aspden, Pavel V Baranov, Ariel Alejandro Bazzini, Elspeth A Bruford, Marie A Brunet, Tristan Cardon, Anne-Ruxandra Carvunis, Claudio Casola, Jyoti Sharma Choudhary, Kellie Dean, Pouya Faridi, Ivo Fierro-Monti, Isabelle Fournier, Adam Frankish, Mark Gerstein, Norbert Hubner, Yunzhe Jiang, Manolis Kellis, Thomas F Martinez, Gerben Menschaert, Pengyu Ni, Sandra Orchard, Xavier Roucou, Joel Rozowsky, Michel Salzet, Mauro Siragusa, Sarah Slavoff, Nicola Ternette, Juan Antonio Vizcaino, Aaron Wacholder, Wei Wu, Zhi Xie, Yucheng T Yang, Robert L Moritz, Eivind Valen, Jonathan Mudge, Sebastiaan van Heesch, John R Prensner, Owen J L Rackham

**Affiliations:** Genome Institute of Singapore (GIS), Agency for Science, Technology and Research (A*STAR), 60 Biopolis Street, Genome, Singapore 138672, Republic of Singapore; Centre for Computational Biology, Cardiovascular and Metabolic Disorders, Duke-NUS medical school, Singapore169857,Republic of Singapore; Cardiovascular and Metabolic Sciences, Max Delbrück Center for Molecular Medicine in the Helmholtz Association (MDC), Berlin 13125, Germany; European Molecular Biology Laboratory, European Bioinformatics Institute (EMBL-EBI), Wellcome Genome Campus, Hinxton, Cambridge CB10 1SD, United Kingdom; Institute of Genetics and Biotechnology, University of Warsaw, 02-106 Warsaw, Poland; Department of Biosciences, University of Oslo, 0316 Oslo, Norway; Princess Máxima Center for Pediatric Oncology, Utrecht 3584 CS, The Netherlands; Oncode Institute, Utrecht 3521 AL, The Netherlands; Department of Pediatrics, Division of Pediatric Hematology/Oncology, University of Michigan Medical School, Ann Arbor, MI 48109, United States; Department of Biological Chemistry, University of Michigan Medical School, Ann Arbor, MI 48109, United States; Institute for Systems Biology, Seattle, WA 98109, United States; Hospital del Mar Research Institute (HMRIB), Barcelona 08003, Spain; Catalan Institute for Research and Advanced Studies (ICREA), Barcelona 08010, Spain; School of Molecular and Cellular Biology, Faculty of Biological Sciences, University of Leeds, Leeds LS2 9JT, United Kingdom; Leeds Omics, University of Leeds, Leeds LS2 9JT, United Kingdom; Astbury Centre for Structural Molecular Biology, University of Leeds, Leeds LS2 9JT, United Kingdom; School of Biochemistry and Cell Biology, University College Cork, Cork T12 K8AF, Ireland; Stowers Institute for Medical Research, 1000 E 50th Street, Kansas City, MO 64110, United States; Department of Molecular and Integrative Physiology, University of Kansas School of Medicine, KS City, KS 66160, United States; HUGO Gene Nomenclature Committee, Department of Haematology, University of Cambridge Clinical School, Cambridge CB2 0PT, United Kingdom; Medical Genetics Service, Pediatrics Department, University of Sherbrooke, J1E 4K8 Sherbrooke, Canada; Cancer Research Institute University of Sherbrooke, IRCUS, J1E 4K8 Sherbrooke, Canada; Centre de Recherche du Centre hospitalier universitaire de Sherbrooke, CRCHUS, J1E 4K8 Sherbrooke, Canada; Univ. Lille, Inserm, CHU Lille, U1192 - Protéomique Réponse Inflammatoire Spectrométrie de Masse - PRISM, Lille F-59000, France; Department of Computational and Systems Biology, University of Pittsburgh School of Medicine, Pittsburgh PA 15260, Pennsylvania; Pittsburgh Center for Evolutionary Biology and Medicine (CEBaM), Pittsburgh PA 15260, Pennsylvania; Department of Ecology and Conservation Biology, Texas A&M University, College Station, TX 77843,United States; The Institute of Cancer Research, London SW3 6JB, UK; School of Biochemistry and Cell Biology, University College Cork, Cork T12 XF62, Ireland; Centre for Cancer Research, Hudson Institute of Medical Research, Clayton, VIC, Australia; Monash Proteomics & Metabolomics Platform, Department of Medicine, School of Clinical Sciences, Monash University, Clayton, VIC 3168, Australia; EMBL-EBI, Wellcome Genome Campus, Cambridgeshire CB10 1SD, United Kingdom; Biozentrum, University of Basel, Basel 4056, Switzerland; Univ. Lille, Inserm, CHU Lille, U1192 - Protéomique Réponse Inflammatoire Spectrométrie de Masse - PRISM, Lille F-59000, France; Institut Universitaire de France, ministère de l’Enseignement supérieur, de la Recherche et de l’Innovation, 1 rue Descartes, 75231 PARIS CEDEX 05, France; European Molecular Biology Laboratory, European Bioinformatics Institute (EMBL-EBI), Wellcome Genome Campus, Hinxton, Cambridge CB10 1SD, United Kingdom; Program in Computational Biology and Bioinformatics, Yale University, New Haven, CT 06520, United States; Department of Molecular Biophysics and Biochemistry, Yale University, New Haven, CT 06520, United States; Department of Computer Science, Yale University, New Haven, CT 06520, United States; Department of Statistics and Data Science, Yale University, New Haven, CT 06520, United States; Department of Biomedical Informatics and Data Science, Yale University, New Haven, CT 06520, United States; Cardiovascular and Metabolic Sciences, Max Delbrück Center for Molecular Medicine in the Helmholtz Association (MDC), Berlin 13125, Germany; Charité-Universitätsmedizin Berlin, 10117 Berlin, Germany; German Center for Cardiovascular Research (DZHK), partner site Berlin, Berlin 13347, Germany; Helmholtz Institute for Translational AngioCardiosciences (HI-TAC), Max Delbrück Center for Molecular Medicine at Heidelberg University, Heidelberg 69120, Germany; Program in Computational Biology and Bioinformatics, Yale University, New Haven, CT 06520, United States; Department of Molecular Biophysics and Biochemistry, Yale University, New Haven, CT 06520, United States; MIT Computer Science and Artificial Intelligence Laboratory, Cambridge, MA 02139, United States; Broad Institute of MIT and Harvard, Cambridge, MA 02139, United States; Department of Pharmaceutical Sciences, University of California, Irvine, Irvine, CA 92617, United States; Department of Biological Chemistry, University of California, Irvine, Irvine, CA 92617, United States; Chao Family Comprehensive Cancer Center, University of California, Irvine, Irvine, CA 92617, United States; BioBix, Lab for Bioinformatics and Computational Genomics, Faculty of Bioscience Engineering, Ghent University, Ghent 9000, Belgium; Program in Computational Biology and Bioinformatics, Yale University, New Haven, CT 06520, United States; Department of Molecular Biophysics and Biochemistry, Yale University, New Haven, CT 06520, United States; European Molecular Biology Laboratory, European Bioinformatics Institute (EMBL-EBI), Wellcome Genome Campus, Hinxton CB10 1SD, United Kingdom; Department of Biochemistry and Functional Genomics, Université de Sherbrooke, Sherbrooke, QC J1H 5N4, Canada; Centre de Recherche du Centre hospitalier universitaire de Sherbrooke, CRCHUS, J1E 4K8 Sherbrooke, Canada; Program in Computational Biology and Bioinformatics, Yale University, New Haven, CT 06520, United States; Department of Molecular Biophysics and Biochemistry, Yale University, New Haven, CT 06520, United States; Univ. Lille, Inserm, CHU Lille, U1192 - Protéomique Réponse Inflammatoire Spectrométrie de Masse - PRISM, Lille F-59000, France; Institut Universitaire de France (IUF), 75000 Paris, France; Goethe University, Institute for Vascular Signalling, Centre for Molecular Medicine, Frankfurt am Main 60590, Germany; CardioPulmonary Institute, Frankfurt am Main 60590, Germany; Yale University Department of Chemistry, New Haven, CT 06520,United States; University of Dundee, Dundee, Scotland DD1 5EH, United Kingdom; European Molecular Biology Laboratory, European Bioinformatics Institute (EMBL-EBI), Wellcome Genome Campus, Hinxton, Cambridge CB10 1SD, United Kingdom; Department of Computational and Systems Biology, School of Medicine, University of Pittsburgh, Pittsburgh, PA 15213, United States; Pittsburgh Center for Evolutionary Biology and Medicine, School of Medicine, University of Pittsburgh, Pittsburgh, PA 15213, United States; Singapore Immunology Network (SIgN), Agency for Science, Technology and Research (A*STAR), Singapore 138648, Republic of Singapore; Department of Pharmacy & Pharmaceutical Sciences, National University of Singapore, Singapore 117543, Republic of Singapore; State Key Laboratory of Ophthalmology, Zhongshan Ophthalmic Center, Sun Yat-sen University, Guangzhou 510060, China; Department of Molecular Biophysics and Biochemistry, Yale University, New Haven, CT 06520, United States; Institute of Science and Technology for Brain-Inspired Intelligence, Fudan University, 220 Handan Road, Shanghai 200433, China; Institute for Systems Biology, Seattle, WA 98109, United States; Department of Biosciences, University of Oslo, 0316 Oslo, Norway; European Molecular Biology Laboratory, European Bioinformatics Institute (EMBL-EBI), Wellcome Genome Campus, Hinxton, Cambridge CB10 1SD, United Kingdom; Princess Máxima Center for Pediatric Oncology, Utrecht 3584 CS, The Netherlands; Oncode Institute, Utrecht 3521 AL, The Netherlands; Department of Pediatrics, Division of Pediatric Hematology/Oncology, University of Michigan Medical School, Ann Arbor, MI 48109, United States; Department of Biological Chemistry, University of Michigan Medical School, Ann Arbor, MI 48109, United States; School of Biological Sciences, University of Southampton, Southampton SO17 1BJ, United Kingdom

## Abstract

Non-canonical (i.e. unannotated) open reading frames (ncORFs) have until recently been omitted from reference genome annotations, despite evidence of their translation, limiting their incorporation into biomedical research. To address this, in 2022, we initiated the TransCODE consortium and built the first community-driven consensus catalog of human ncORFs, which was openly distributed to the research community via Ensembl-GENCODE. While this catalog represented a starting point for reference ncORF annotation, major technical and scientific issues remained. In particular, this initial catalog had no standardized framework to judge the evidence of translation for individual ncORFs. Here, we present an expanded and refined catalog of the human reference annotation of ncORFs. By incorporating more datasets and by lifting constraints on ORF length and start codon, we define a comprehensive set of 28 359 ncORFs that is nearly four times the size of the previous catalog. Furthermore, to aid users who wish to work with ncORFs with the strongest and most reproducible signals of translation, we utilized a data-driven framework (i.e. translation signature scores) to assess the accumulated evidence for any individual ncORF. Using this approach, we derive a subset of 10 127 ncORFs with translation evidence on par with canonical protein-coding genes, which we refer to as the primary set. This set can serve as a reliable reference for downstream analyses and validation, with a particular emphasis on high quality. Overall, this update reflects continuous community-driven efforts to make ncORFs accessible and actionable to the broader research public, and further iterations of the catalog will continue to expand and refine this resource.

## Graphical Abstract

**Graphical Abstract:**
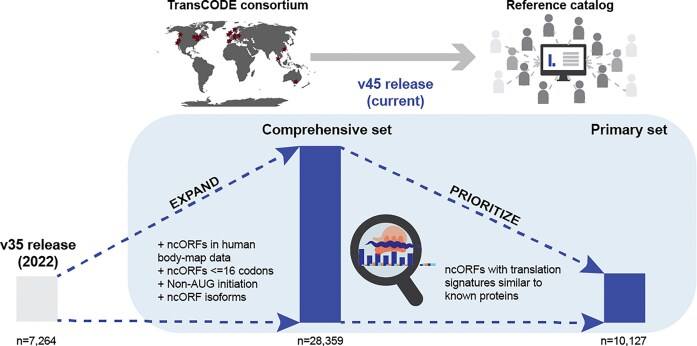

## Introduction

Since the creation of the Ensembl-GENCODE (hereafter GENCODE) project 20 years ago [1, 2], gene annotations have been broadly divided based on whether a given gene is *protein-coding* or *non-coding*. Today, the canonical set of coding sequences (CDS) produced by GENCODE is a key resource for understanding the translated portion of the transcriptome. However, an increasing understanding of the prevalence of translation outside the set of canonical protein annotations has revealed the presence of additional functional elements. Here, perhaps no development in genome technologies has been more provocative than the adoption of ribosome profiling (Ribo-seq) [3], which has led to the identification of thousands of translated short unannotated open reading frames (typically 100 or fewer codons) [4–12]. We refer to these as translated non-canonical ORFs (ncORFs), specifically meaning ORFs with translation evidence that fall outside GENCODE’s standard protein-coding annotations. Hereafter, we omit the term “translated” for brevity. In some cases, ncORFs are discovered to encode *bona fide* “microproteins” that can then be classified as canonical [13], leading to newly annotated protein-coding genes such as *MTLN* [14], *MRLN* [15], and *MIEF1-uORF/L0R8F8* [16–19]. However, many ncORFs may not meet the evolutionary constraints expected of canonical proteins [13, 20], yet potentially encode species-specific functional proteins [11, 21] or, alternatively, serve regulatory roles through their translation [22–25]. NcORF translation has been shown to occur broadly under physiological conditions, but there is great interest in the identification of ncORF translation products that may be recurrently or uniquely associated with specific cellular or pathological states [26–32]. Their widespread yet poorly understood nature highlights the need for standardized annotation to enable broader investigation by the scientific community.

Ultimately, the annotation of translated ncORFs depends on the accumulation and analysis of Ribo-seq data, the development of a new, more sophisticated classification schema for translation, and the additional inclusion of orthogonal protein evidence. To this end, in 2022, we initiated the global TransCODE Consortium, in collaboration with GENCODE [33], HGNC (HUGO Gene Nomenclature Committee) [34], UniProtKB (UniProt Knowledge Base) [35], PeptideAtlas [36], and other leading academic labs. This consortium combines expertise in annotation, Ribo-seq, gene evolution, non-coding RNA function, and mass-spectrometry proteomics. The result of Phase I of TransCODE was an initial GENCODE-supported catalog of 7264 ncORFs [20] previously identified using Ribo-seq as well as guidelines on how to interpret Ribo-seq and other experimental data nominating such ncORFs for annotation [22].

However, community-wide usage of these 7264 ncORFs has revealed both their utility as well as their limitations. It has become clear that this first catalog of ncORFs contains blind spots, making an updated catalog essential for the community. Several such blind spots resulted from the criteria used for ncORF inclusion, which were employed at that time for practical rather than biological reasons. For example, the 7264 ncORFs only include candidates that are: (i) >16 codons in length, (ii) initiated at AUG start codons, and (iii) the longest isoform in situations where multiple ncORFs share a large part of their amino acid sequence (≥90%). Yet, such filters likely hinder research on some ncORFs, particularly small ones. Indeed, it is known that ncORFs as small as two codons (i.e. “start–stop” or “minimal” ncORFs) [37] can function as regulatory elements, and recent studies in human tissues and cell lines have demonstrated widespread translation of ncORFs below our previous size cutoff [11, 12]. Moreover, translation products of ncORFs as short as eight codons can be presented on major histocompatibility complex molecules [27]. Similarly, non-AUG translation initiation events are increasingly recognized as biologically relevant, even within well-annotated protein-coding genes [38, 39, 40], with increasing evidence that they are especially common within 5′ untranslated regions (5′ UTRs) [12, 20]. Additionally, alternative splicing, the use of alternative initiation codons, or sequence variation may be specific to disease states [21, 26] or even to different stages of the cell cycle [41], making it possible to have multiple ncORF isoforms. Altogether, for the field of ncORF research to continue to grow, it is now critical to develop reference annotations of ncORFs further.

Here, we present an updated reference catalog of translated ncORFs detected by Ribo-seq based on GENCODE v45. As with the Phase 1 workflow, our goal was to use Ribo-seq data to map ncORFs to annotated transcripts; however, in this updated catalog, we incorporate additional studies made available since the production of our first catalog and remove the restrictive filters (as described above, Supplementary Fig. S1a and b). We first generate a “Comprehensive set” that substantially increases the number of ncORFs called from Ribo-seq from 7264 (Phase I) to 28 359. The new catalog adds thousands of ncORFs that were initially excluded from the first catalog, as well as ncORFs from the human body map translation project, extending the coverage of our resource to 11 additional primary human tissues and cell types [12]. We emphasize that “*Comprehensive”* refers to the inclusive approach taken in incorporating ncORFs into this set, as opposed to the sense that the catalog may be biologically complete (which remains hard to assess). As another advancement over our previous work, we specifically designate ncORFs with robust evidence of translation using two large pooled human Ribo-seq datasets into a “Primary set” of ncORFs. To do so, we utilize a standardized assessment of ncORFs using translation signature scores that act as quality-control metrics, namely, “P-sites in frame (PIF)”, “Uniformity”, and “Dropoff” scores (previously used in the human body map translation project [12, 42]). Based on these scores, we denote a subset of 10 127 out of 28 359 ncORFs as the “Primary” set, as these ncORFs have translation signature scores in the same range as canonical protein-coding sequences. The purpose of this Primary set (as opposed to the Comprehensive set) is to allow users to focus their analyses on ncORFs with the highest degree of translation evidence currently available. To ensure that these ncORF annotations can be widely applied across biomedical research, both sets are available at https://www.gencodegenes.org/pages/riboseq_orfs/. 

## Materials and methods

### Collecting ncORFs from multiple datasets and mapping to GENCODE v45

We previously initiated the first catalog by selecting seven distinct Ribo-seq ncORF datasets from a range of human studies, each of which has been pivotal for genome-wide ncORF identification using Ribo-seq over the past decade [20]. Here, we included these seven datasets plus two new Ribo-seq ncORF datasets that were published in 2022 [12] and 2023 [11] (Supplementary Table S1).

**Figure 1.**
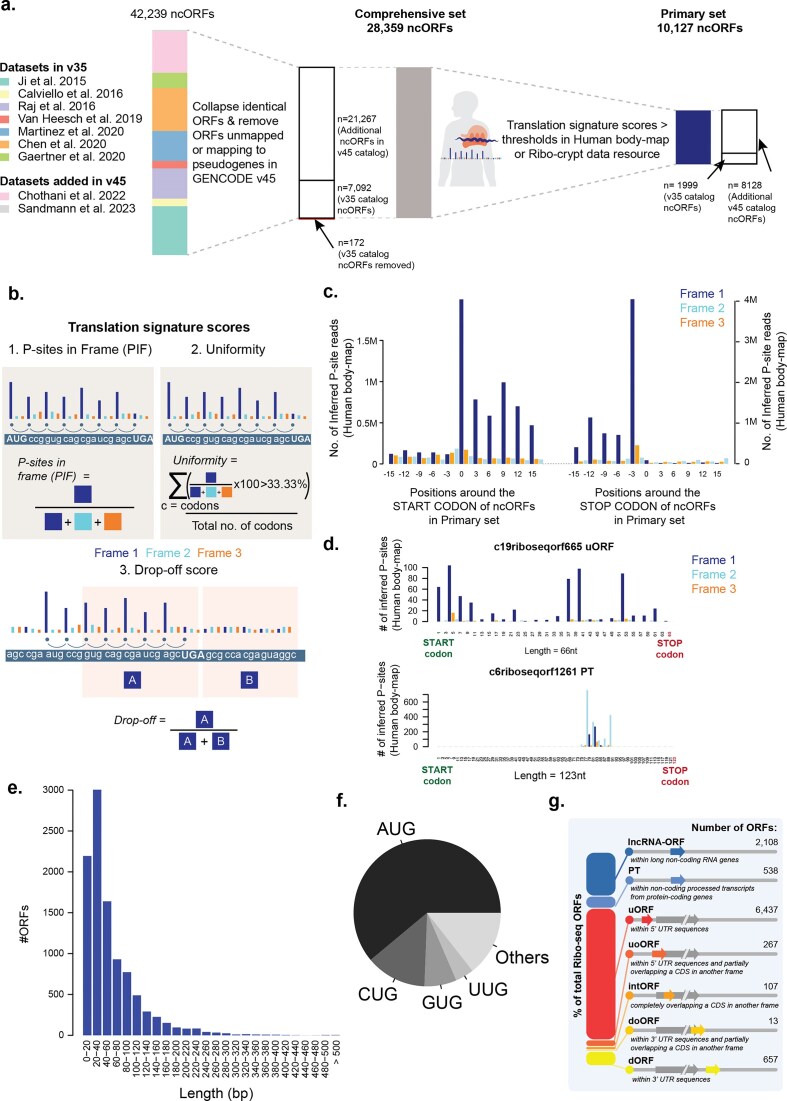
A Phase 2 GENCODE catalog for ncORFs using translation signatures. (**a**) Sankey plot based flow chart showing number of ncORFs in comprehensive set and selection to primary set. NcORFs were collected from nine studies [4–12] listed as Datasets, without any hard filters on length or start-codon, followed by obtaining a comprehensive set by collapsing identical ncORFs and removing ncORFs not mapping to GENCODE v45 or mapping to pseudogenes. Translation signature scores applied on each ncORF using human body-map or RiboCrypt to obtain *Primary* set. (**b**) Translation signature scores and its quantification. This included three metrics to define translation signature presence in each ncORF. *PIF* quantifies the proportion of Inferred P-sites in the translating frame with respect to the total inferred P-sites in the ncORF. *Uniformity* quantifies the percentage of codons in the ncORF that have > 33.33% inferred P-sites in the translating frame. *Drop-off score* quantifies the proportion of inferred P-sites before the stop-codon with respect to the total inferred P-sites in the translating frame in a 30bp window around the stop codon. (**c**) A bar plot showing an aggregated P-site profile (using the human body-map) around the start- and stop-codons of the *Primary* set of ncORFs (**d**) A bar plot showing P-site profile of two selected Ribo-seq ncORFs with high and low translation signature scores. (**e**) A histogram showing the length distribution of *Primary* set ncORFs. (**f**) A pie chart showing the distribution of start-codons identified for the *Primary* set ncORFs. (**g**) A stacked bar chart showing numbers of *Primary* set ncORFs across different ncORF types.

When available, we retrieved exonic coordinates and sequences for these ncORFs. For datasets based on the older human genome assembly (GRCh37/hg19), we converted ORF coordinates to GRCh38/hg38 using UCSC Liftover. We compiled a total of 42 239 ncORFs. All translated ORF sequences were remapped to the Ensembl Release v.111 transcriptome (equivalent to GENCODE v45), collapsing identical genomic ncORF sequences. This resulted in 30 103 unique genomic sequences after excluding 1452 ORFs that could not be matched to any transcript and 10 684 identical ncORF regions. Our selection was limited to ncORFs found in lncRNAs, non-coding RNA transcripts within known protein-coding genes, alternative reading frames of CDS annotated within known protein-coding genes, and untranslated regions (UTRs) of coding transcripts annotated within known protein-coding genes. Therefore, we excluded 1744 ncORFs partially or totally overlapping annotated CDSs in the same frame (protein-coding or nonsense-mediated decay) or pseudogenes in any frame, as was done for the previous catalog [20]. These exclusions were necessary, as the ncORF datasets were based on older transcriptome versions, and newly annotated protein-coding sequences and pseudogenes are now available in GENCODE v45. Of 80 v35 catalog ncORFs that are now reclassified as CDSs, 49 previously overlapped incomplete protein-coding CDSs annotated with missing 5′ or 3′ regions, but we now completely classify these cases as CDS. Pseudogenes were removed because not all the original studies included only uniquely mapped reads, which could have led to false identifications of such sequences, although we acknowledge that many pseudogenes are known to be expressed and translated.

In total, we generated a Comprehensive set of 28 359 ncORFs. Of these, 7092 ncORFs were already part of the first catalog, and 21 267 were newly added. Unlike the first catalog, we did not apply any minimum length filter or exclude non-AUG start codons. Non-AUG ncORFs were predicted in five of the nine considered studies, with the exception of those using RiboTaper or ORFquant, which only annotated AUG-initiated ncORFs [4, 6, 9, 11]. Notably, in [7], ncORFs without an identifiable AUG start codon were defined from stop codon to stop codon, facilitating the inclusion of non-AUG-initiated ncORFs. Because of this, we acknowledge that some of the non-AUG ncORFs presented here may have inaccurate initiation site annotations.

### Transcript assignment and classification of identified ncORFs

The vast majority of ncORFs overlapped several transcript models within a given gene and could not be uniquely mapped to a single host transcript. To resolve these ambiguities, we assigned the ncORF to the main isoform selected using MANE [43]. For the rest of the cases that could not be assigned to the MANE Select isoform, we chose the isoform with the highest APPRIS [44] score as the most probable isoform translating the ORF. In cases where multiple transcripts had comparable APPRIS scores, we further examined the Ensembl transcript support level scores and selected the one with the highest support, prioritizing protein-coding transcripts over non-coding ones. Of note, transcripts annotated as readthrough were given the lowest level of support and were assigned to an ncORF only when no other compatible isoform was available. All potential Ensembl transcript and gene IDs associated with each ORF, as well as the selected host transcripts, are detailed in Supplementary Table S2. While we made sure that the ncORFs do not partially or totally overlap the amino acid sequences of any annotated CDSs in the same region, it is possible that future releases of transcript annotations will include new models, and at least some ncORFs may be reannotated as new CDS extensions resulting from alternative splicing [45].

NcORFs were categorized into seven distinct types exactly as defined for the first catalog [20], i.e. based on the biotype of the assigned host isoform and the ORF position relative to known canonical protein-coding sequences.

lncRNA-ORFs: ncORFs found on long non-coding RNA genes.

PT-ORFs: ncORFs encoded by non-coding transcripts from protein-coding genes.

Upstream ORFs or uORFs: encoded within 5′ UTR sequences.

Upstream overlapping ORFs or uoORFs: encoded within 5′ UTR sequences and partially overlapping an already annotated CDS downstream in an alternative frame.

Internal ORFs or intORFs: completely overlapping within an already annotated CDS in an alternative frame.

Downstream ORFs or dORFs: encoded within 3′ UTR sequences.

Downstream overlapping ORFs or doORFs: encoded within 3′ UTR sequences and partially overlapping an already annotated CDS in an alternative frame.

Lastly, as a further advancement in this catalog, we now modify our GENCODE naming of these ORFs. We have decided not to proceed with the dual system used previously, e.g. c1riboseqorf1/c2norep2. This decision was made because we consider that ORFs should not be named according to their reproducibility, e.g. c2norep2, as this could potentially change. Therefore, for this catalog, we now use a simplified system based around only “c1riboseqorf1” and “c1riboseqorf2”. We keep the names of ncORFs from the previous catalog where appropriate, i.e. for those that were called as replicated, and subsequently continuing numbering “upwards” to assign (i) new names for ncORFs found in the previous catalog that were not replicated (i.e. previously annotated as “norep”) and (ii) names for novel ncORFs not included in the first catalog. The “norep” names that were used in the first catalog are also listed in the new datafile, as “legacy_names_v35” to allow comparison with the previous catalog. We anticipate that a standardized nomenclature system classifying ncORFs will be devised in due course.

### Creating test datasets by pooling Ribo-seq data for primary set selection

In this study, we used two pooled Ribo-seq data resources. First, we obtained the previously published human body-map dataset [12], generated using the workflow as previously described. Read alignment was carried out using STAR [46] and only uniquely mapped reads were retained. From these, only read lengths between 27 and 30 nucleotides that showed >60% overall 3-nt periodicity when assessed in annotated protein-coding genes were selected to generate the P-site read files. P-site files from individual samples were then combined to create the pooled body map dataset, which was used to quantify translation signature scores. This resulted in a 1.3 billion P-site dataset across 11 cell types and tissues with an overall 3-nt periodicity of 85%.

Second, we used the Ribocrypt data repository. Metadata for publicly available ribosome profiling datasets were obtained from the Ribosome Data Portal [47]. Sequencing libraries corresponding to ribosome profiling samples were downloaded and processed with massiveNGSpipe (https://github.com/rc-biotech/massiveNGSpipe), as outlined in Swirski *et al.* 2025 (manuscript in submission). Briefly, raw FASTQ files were downloaded from the Amazon AWS NCBI SRA mirror (https://registry.opendata.aws/ncbi-sra). Adapters and barcodes were detected with a custom function. Their removal and length trimming were performed with fastp [48], using a minimum read-length cutoff of 20 nt. Identical reads were then collapsed, with copy number encoded in the read name, to reduce computational burden. Subsequently, reads were aligned to the human reference genome (hg38) with STAR [46], using the following non-default parameters: min.length = 20, mismatches = 3, trim.front (5′) = 0, max.multimap = 10, alignment.type = “Local”. P-site positions were inferred with the shiftFootprintsPerExperiment function from ORFik [49]. For each library and read length, this procedure estimates the distance between the 5′ end of the read and the P-site. Only read lengths exhibiting clear triplet periodicity, as detected by a Fast Fourier Transform (FFT)-based algorithm, were subjected to the P-site offset detection algorithm described in detail by Tjeldnes *et al.* [49]. The human merged track was then created using massiveNGSpipe::pipeline_merge_org function. Only uniquely aligned reads were used to construct the merged track.

Ribo-seq coverage profiles across all included genes can be visually inspected under the link:

https://ribocrypt.org/?dff=all_merged-Homo_sapiens_modalities&frames_type=columns&kmer=1&colors=Color_blind&unique_align=TRUE.

In total, for Ribocrypt data, 3,892 human ribosome profiling libraries make up the merged track, amounting to 218 billion individual reads and 30.42 billion bona-fide footprints (unique alignments from periodic read lengths that do not map to rRNA).

### Quantifying translation signature scores for ncORFs

We quantified translation signature scores for each ncORF in the Comprehensive set to test for evidence of translation in two test datasets generated by pooling Ribo-seq resources as described above.

Translation signature scores include three metrics as previously described ([12], [42]), namely (i) PIF, (ii) Uniformity, and (iii) Drop-off. P-sites in frame were calculated as the proportion of inferred P-site reads in the translating frame of the ncORF to the total inferred P-site reads in the ncORF. For the Uniformity calculation, each codon was tested for the proportion of P-site reads in Frame 1 to the total reads in the codon. The number of codons that had >33% of reads in Frame 1 were considered as having evidence for translation, and the proportion of codons that shows translation to the total number of codons was considered as the Uniformity. Last, for Drop-off score, a 15 bp transcript flanking window on either side of the stop codon was selected. P-site reads before and after were quantified, and Drop-off was calculated as the ratio of reads before the stop codon with respect to the total reads in both before and after flanking regions. These three scores were calculated for each ncORF in the Comprehensive set independently in each of the test datasets. These scores were also quantified previously [12] for known protein-coding ORFs to identify ncORFs with similar translation signatures. A normal distribution was fitted to the score distributions of annotated protein-coding ORFs, and thresholds were defined based on the 95th percentile for PIF and Uniformity, and the mean value was used for Drop-off score. For the human body-map dataset, the thresholds were determined as 75.89% for PIF, 71.3% for Uniformity, and 92% for Drop-off. For the Ribocrypt dataset, the thresholds were determined as 51.12% for PIF, 87.79% for Uniformity, and 88% for Drop-off scores, respectively. The ncORFs in the Comprehensive set that passed these thresholds in either of the test datasets were considered to have high evidence for translation and selected for the Primary set. The ncORFs in the Primary set and the Comprehensive set were ranked for the Uniformity scores and the top 10 ncORFs from Primary set and the bottom 10 ncORFs from Comprehensive set were selected for visualization (P-sites per million or PPM <1 were skipped in the selection. PPM calculation described in the next section). The scores were able to select for individual ncORFs, which showed clear periodicity throughout the length of the ncORFs, thus discriminating ncORFs from ncORFs with just a single read stack or discontinuous periodicity or periodicity in the wrong frame (Supplementary Fig. S2 and  Supplementary File S1).

### Isoforms of ncORFs and overlap across studies

We compiled ncORFs from various studies, each using different datasets and methodologies. While identical ncORFs were treated as unique instances, we also identified many cases of ncORFs sharing part of their codon sequences. Therefore, ncORFs sharing any codon sequence were classified as ncORF isoforms. All isoforms were included in both the Comprehensive and Primary sets. A description of all and primary ncORF-overlapping isoforms is available at Supplementary Tables S5 and S6, respectively.

### Testing limitations in Primary set

P-sites per million (PPM) values were calculated by normalizing ORF P-site counts by the ORF length and scaling to per-million total normalized counts. PPM values were quantified for each ncORF and annotated protein-coding ORF, and PPM >1 values were considered as a threshold for expression. NcORFs in the Comprehensive set and Primary set were split into length bins of 20 nucleotides (nt) each until 500 nt and into ncORF type categories such as uORF, dORF, uoORF, doORF, lncRNA-ORF, PT-ORF, and scores for such length bins and categories were presented using boxplots. Proportion of ncORFs in each bin and category was quantified and a chi-square test was carried out to test if the proportion of ncORFs that passed the PIF and Uniformity scores changed for ncORFs that are less than or more than 100 nt. PIF and Uniformity scores were tested in annotated ORFs across the same length bins as ncORFs for reference (Supplementary Fig. S4f). Genes with length <100 amino acids were selected, and distributions of PIF, Uniformity and Drop-off were plotted for a randomly selected transcript per gene. P-site profiles were plotted for known microproteins using human body map, and RiboCrypt data resource and translation signature scores were calculated for each microprotein (Supplementary Fig. S5).

**Figure 2.**
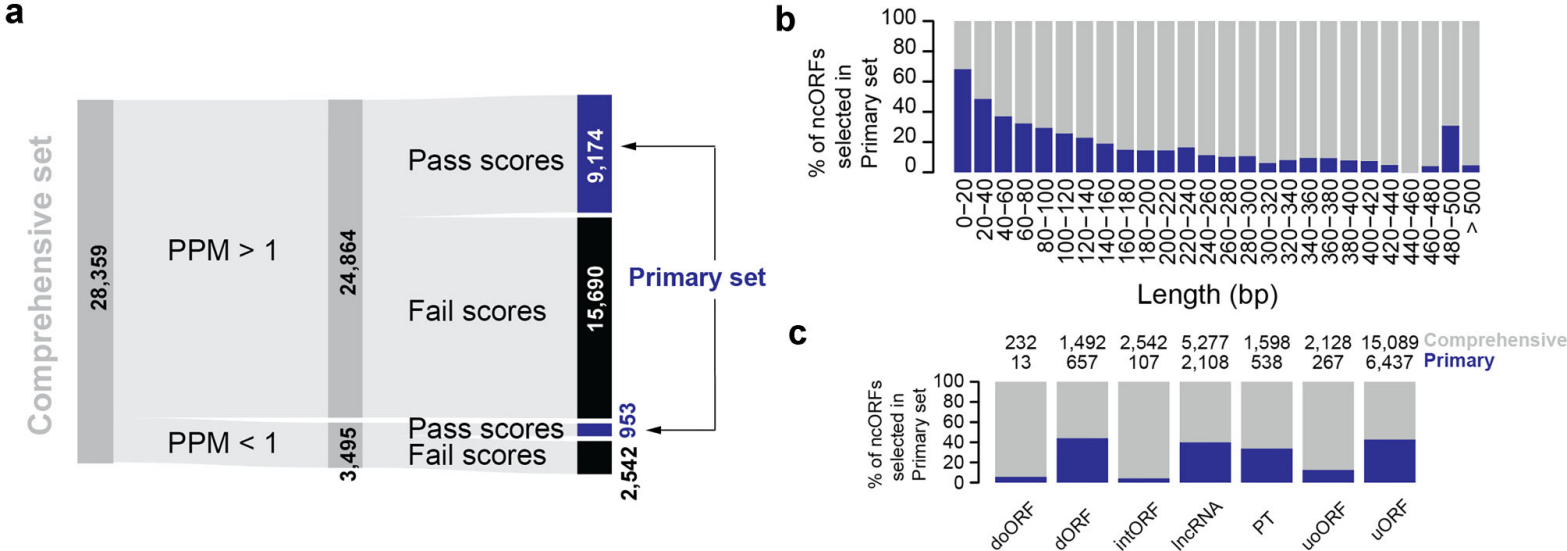
Coverage of translation signatures in Comprehensive vs Primary set. (**a**) A Sankey plot showing the number of ncORFs in the comprehensive set and corresponding ORFs that have P-sites per million (PPM) >1 in the pooled human body-map Ribo-seq data followed by the number of ncORFs that pass the translation signature scores. (b, c) Stacked barplot showing the proportion of ncORFs passing thresholds across length bins (**b**) and within each ncORF type (**c**). Gray: Comprehensive set ncORFs, blue: Primary set.

## Results

### An expansion of the ncORF catalog

The new catalog of ncORFs is mapped to GENCODE v45 and is now referred to as the “v45” catalog. It builds upon our initial release in July 2022, which was mapped to GENCODE v35 [20] (see Table 1). The v45 catalog incorporates datasets from the same seven published studies as used for the first catalog, and according to the same selection principles (see Methods for details) includes two additional human Ribo-seq ncORF datasets that were published in 2022 [12] and 2023 [11] (Supplementary Table S1), leading to a 62% increase in ncORF-calling samples and adding 11 cell-type or tissue coverage. First, Chothani *et al.* [12], carried out an extensive ncORF human translation body map study, generating more than a billion inferred P-site reads using 167 Ribo-seq samples. These samples covered six primary human cell types (atrial fibroblasts, coronary artery endothelial cells, umbilical vein endothelial cells, hepatocytes, vascular smooth muscle cells, embryonic stem cells) and five human tissues (brain, adipose tissue, heart, skeletal muscle, kidney), resulting in the identification of 7767 ncORFs. Second, we incorporated 221 experimentally interrogated ncORFs reported by Sandmann *et al.* [11], previously not included, as each was 16 codons or fewer in length. The v45 catalog also takes a more inclusive approach to ncORF identification; we now include ORFs of 16 codons or less, those initiated by non-AUG codons, and all identified isoforms of each ncORF (Supplementary Fig. S1a and b).

**Table 1.** Comparison of v35 and v45 ncORF catalog

	v35 catalog (2022)	v45 catalog (2025)	
		Comprehensive set	Primary set
Total ncORFs	7264	28 359	10 127
Number of studies included	7	9	9
Number of Ribo-seq samples included	139	226	226
Overlap of v35 ncORFs	-	7092	1999
Length restriction	Yes (>16 codons)	No	No
Start-codon restriction	Yes (AUG only)	No	No
Isoforms included	Partially (clustered isoforms ≥90% shared codon sequence)	Yes	Yes
Standardized metrics applied (translation signature scores) using pooled human-body map or RiboCrypt data	No	No	Yes

In total, we identified 28 359 ncORFs that we refer to as the “Comprehensive set” of our v45 catalog (Fig. 1a and Supplementary Table S2). We retained most of the ncORFs (7092 out of 7264) from the first catalog, excluding 172 prior ncORFs because (i) they no longer mapped to GENCODE transcripts in v45 due to changes in transcript annotations, which now place these ncORFs partially or entirely in intergenic or intronic regions (*n* = 65), (ii) are now annotated as known proteins [13, 20] (*n* = 25), (iii) have been reassessed as extensions of known proteins (*n* = 80), or (iv) map to pseudogenes (*n* = 2) (Supplementary Fig. S1c and Supplementary Table S3). Building upon this, the Comprehensive set also includes: (i) 3124 additional isoforms (due to alternative splicing or alternative translation initiation) of ncORFs that were present in the first catalog and (ii) a further 18 143 ncORFs that do not overlap with any of the ncORFs from the first catalog (Supplementary Fig. S1d).

### Data-driven discrimination of a Primary set of ncORFs for improved end-user usability

In gene annotation, filtered subsets help users navigate large transcript collections, such as the 19 252 Matched Annotation from NCBI and EBI (MANE) transcripts within the 252 989 total transcripts in GENCODE v45 [43]. While MANE prioritizes functionally relevant, well-supported models, ncORFs currently lack equivalent functional information. Therefore, we rely on data-driven metrics to identify a filtered set of ncORFs. In our initial catalog (v35) [20], we tested for replication of ncORFs across multiple studies as a proxy for detection reliability (Supplementary Fig. S1e-g). However, this approach is constrained by substantial variability in both the quality of the individual Ribo-seq dataset as well as the nature and performance of computational workflows deployed. These issues are further confounded by the relative sparseness of Ribo-seq reads across an ncORF when using single samples or smaller Ribo-seq datasets [13, 20, 50]. To address these inconsistencies, here we define a Primary set by using two large pooled Ribo-seq resources [The Human Bodymap [12] and RiboCrypt (https://ribocrypt.org/)] as a way to assess the translational signatures, using three data-driven metrics. By ensuring that a primary ncORF satisfies these metrics, irrespective of the method of identification, we can ensure a more robust and consistent filtering strategy.

To construct the Primary set, we apply filters based on features derived from Ribo-seq data, selecting ncORFs whose translation signatures closely resemble those of canonical protein-coding genes. As a result, to standardize the evidence for ncORFs obtained from different studies, we independently assessed each ncORF to identify a subset of ORFs featuring the same degree of experimental evidence as observed for canonical CDSs. Specifically, we used Ribo-seq data-derived P-site profiles using the pooled human body-map data [12, 42] and pooled RiboCrypt data (https://ribocrypt.org/) to assess each individual ncORF in the Comprehensive set using previously designed metrics [12, 42]: (i) “PIF”, which determines the proportion of inferred P-site reads that are found in the translating frame; (ii) “Uniformity”, which quantifies the proportion of codons within the ncORF that have >33.33% reads in the translating frame; and (iii) the “Drop-off score”, which quantifies the proportion of P-sites in-frame before the stop codon to the total P-sites in-frame within the 30 bp window before and after the stop codon (Fig. 1b; see the “Materials and methods” section for details). An ncORF from the Comprehensive set was included in the Primary set if it passed the determined threshold in either the human body map or RiboCrypt data. Thresholds were determined for RiboCrypt dataset using annotated CDS with the same methodology as previously determined for the human body-map dataset [12] to identify ncORFs with similar translation signatures to annotated CDSs. As a result, we compiled a Primary set of 10 127 ncORFs (from the 28 359 in the Comprehensive set) (Fig. 1a and Supplementary Table S2; comparison to v35 release shown in Fig. 1a, Table 1, and Supplementary Fig. S1h and i). Upon aggregating P-sites around the start and stop of the ncORFs, we found clearer translation signatures for the Primary set as compared to the remainder of the Comprehensive set (Fig. 1c and d, Supplementary Figs S1j and S2, and Supplementary File S1).

### ncORFs in the Primary set with alternative features

Of the 10 127 ncORFs in the Primary set, 5981 ncORFs (59.0%) are ≤16 codons in length (Fig. 1e and Supplementary Fig. S1h and i), and 3943 ncORFs (38.9%) start from non-AUG initiation codons (Fig. 1f), two thresholds applied in our initial v35 catalog. In the Primary set, we found that almost two-thirds of the identified ncORFs are upstream ORFs (uORFs, *n* = 6437, 63.6%, Fig. 1g) followed by ORFs in long non-coding RNA (lncRNA) genes (lncRNA-ORFs, *n* = 2108, 20.8%) (Potential explanations of these observations are discussed below in the Limitations section). For example, the Primary set includes the 4-codon-long uORF c22riboseqorf449 in *ATF4*, which is known to influence downstream CDS translation upon stress or disease [51]. The Primary set also includes 114 “minimal ORFs” that only contain a start and a stop codon. Such minimal ORFs could serve as regulatory elements [37] and are thus an important inclusion for reference annotations [52]. Within the Primary set, we found that 61.1% of ncORFs initiate at AUG codons, 13.3% at CUG, and 7.0% at GUG, similar to what has been previously reported by others [53] (Fig. 1f).

Next, we sought to define ncORF isoforms in the Primary set that can arise via alternative splicing and/or alternative initiation. These processes have historically been challenging to resolve in Ribo-seq data due to the sparsity of Ribo-seq coverage throughout the length of individual ncORFs [22], as well as complexities in the underlying transcript models. Furthermore, it is also expected that both processes can be subjected to differential expression, i.e. across cell types and different biological conditions. Within the Primary set, we identified 3303 ncORF isoforms that partially share the amino acid sequences of other ncORFs within the same set (Supplementary Fig. S3a), representing 32.6% of the total set. Of these, 185 and 2921 ncORFs shared identical start and stop positions, respectively, with other ncORFs (Supplementary Fig. S3a), while an additional 168 exhibited overlaps at either the start or stop codon of other ncORFs. Notably, only 332 ncORFs exhibited an overlap of ≥90% with other ncORFs, indicating that most ncORF isoforms contributed substantial stretches of unique codon sequences. We also identified 1259 non-AUG-initiating ncORFs as isoforms of AUG ncORFs and 3281 non-AUG-initiating ncORFs that do not have other AUG-initiating ncORFs included in the Primary set. For instance, *LMBRD2* encodes two overlapping uORFs with partially shared codon sequences that are translated into two small ncORF products of 39 and 42 codons, respectively, in the complete absence of a nearby AUG (Supplementary Fig. S3b). As another example, the *MIR1915HG* lncRNA contains two different ncORF variants starting with two alternative non-AUG and AUG codons that each display evidence of translation throughout their length (Supplementary Fig. S3c–e).

### Assessment of gaps in the Primary set

In this resource, we have separated the process of ncORF identification (a process that is handled in the source manuscript of ncORF) and the creation of translation signatures (which are computed in this resource). The reason for this choice is that without a community-agreed pipeline for ncORF calling, we believe that independently scoring each ncORF using the same metrics applied to the same extensive Ribo-seq datasets provides a reliable way to get a standardized Primary set. However, the mismatch between the samples from which ncORFs were called and the samples from which the scores were calculated might create a potential blind spot for the resource. To address this potential blind spot in our workflow, we tested whether the human body map or Ribocrypt data provided sufficient coverage to test all ncORFs, regardless of their source tissue or cell type. Evaluating each ncORF for their expression levels, we found that 87.7% of ncORFs in the Comprehensive set (24 864 out of 28 359) had a PPM >1 in either of the two resources, enough to confidently allow for their analysis. In contrast, 3495 ncORFs had low P-site coverage (PPM <1); despite their low expression, 27.3% still passed the translation signature scores and met the criteria for inclusion in the Primary set (Fig. 2a). However, it is important to note that some of the remaining ncORFs with low coverage that did not meet the criteria may exhibit high translation scores in other tissues or cell lines that were not analyzed in this study.

Furthermore, we tested whether ncORF length and type (Fig. 1e and g; see the “Materials and methods” section for details) influenced their chances of being included in the Primary set using the abovementioned Ribo-seq quality metrics. We did so because we reasoned that metrics like Uniformity could be biased in favor of short ncORFs, where ribosome footprint coverage across a small number of codons would still result in good uniformity. Similarly, we anticipated that ncORF types that overlap other ncORFs in alternative reading frames would score poorly on each of the three metrics. Indeed, we found that smaller ncORFs perform relatively well compared to longer ncORFs, indicating a length-dependent reduction in ncORF performance for the PIF and Uniformity metrics (Fig. 2b and Supplementary Fig. S4a and b). For example, 63.48% (12 303 out of 19 382) of ncORFs <100 nucleotides in length had a PIF score above the threshold in either of the resources, compared to 37.22% (3341 out of 8977) of ncORFs >100 nucleotides (Chi-square test, *P*-value 2.87 × 10^−117^). Second, we found that ncORFs overlapping with CDS sequences in a different reading frame, such as uoORFs, doORFs, or intORFs, had limited representation in the Primary set as the scores assume a single ORF is translated in the given region (Fig. 2c and Supplementary Fig. S4 c, d, and e). Together, this shows that longer ncORFs, overlapping ORFs, and internal ORFs may be underrepresented in the Primary set, and investigators interested in these ncORF types can source these from the Comprehensive set instead.

### Availability of the new GENCODE catalog

The Comprehensive and Primary sets are available at https://www.gencodegenes.org/pages/riboseq_orfs/. A track hub is available at https://ftp.ebi.ac.uk/pub/databases/ensembl/riboseq/TransCODE_GENCODEv45_riboseqORF_catalog/TransCODE-GENCODE_v45_RiboSeqORFs.hub.txt. A UCSC Browser session with appropriate Gencode comprehensive annotation is available at https://genome-euro.ucsc.edu/s/jackt/Phase2%2DTransCODE. Regardless of their current level of supporting data, we emphasize that every ncORF in the Comprehensive set has been published as being translated in at least one of the source studies and thus serves as a valuable reference for tracking and comparison in ncORF discovery, even if absent from the Primary set. We anticipate that some Comprehensive set ncORFs may gain additional supporting data over time, allowing them to be reclassified into the Primary set in future updates.

## Discussion

Despite significant advances in our understanding of the human genome, gene and ORF annotations remain a work in progress. Redundancy in independent efforts for generating ncORF catalogs, as well as low overlap across published sets, has highlighted the need for a unified reference annotation. This need has been further amplified by ongoing efforts to characterize potential protein-coding genes from ncORF catalogs [13, 54]. Here, we have assembled an updated catalog of human ncORFs, seeking to address several current gaps in ncORF annotation. We now provide an expanded Comprehensive set of 28 359 ncORFs, aggregated from the several large-scale ribosome profiling studies, without any length or start-codon filters (considering all possible start codons reported in their respective studies), intended for efforts aiming to assess the ncORF search space in an inclusive manner. Additionally, we denote a Primary set of 10 127 ncORFs that have clear translation signatures in at least one large pooled human dataset, using a combination of PIF, Uniformity, and Drop-off scores. This subset may be useful for analyses where limiting false positives is critical, and we expect these highly supported ncORFs will serve as a “gold standard” set. We view the value of this updated catalog as threefold: (i) a unified resource for researchers; (ii) a high-quality Primary set that may serve as a field reference; and (iii) the development of standardized metrics to quantify translation evidence. These features may prove valuable for worldwide efforts to characterize the biological function of ncORFs [8, 26, 55–57]; the protein-coding potential of ncORFs [13], including initiatives such as the Understudied Proteins Initiative [58] and iMOP [59]; the evolutionary dynamics of ncORFs [11, 21, 60]; the tissue- and disease-specificity of ncORFs; and the potential for nucleotide variants to impact ncORFs in human health and disease.

Potential users of this updated ncORF catalog should note that the Primary set has many very short ncORFs; 59.0% of all annotated ncORFs are 16 codons or shorter. In part, this is to be expected from both a technical and biological perspective. Shorter ncORFs can achieve high PIF and Uniformity scores with fewer base pairs tested, and detection of shorter ncORFs may also be expected, given that longer ncORFs are more likely to trigger nonsense-mediated decay [61]. Many of these short ncORFs are found in the 5′ UTRs of mRNAs, and because mRNAs have higher transcript expression overall compared to lncRNAs, this may increase detection sensitivity for uORFs and uoORFs [62]. Additionally, 5′ UTRs are known hotspots for the emergence of microproteins and play a central role in translational regulation, including canonical protein expression control [63, 64]. An inverse relationship between uORF length and translational reinitiation efficiency has also been observed [23]; and recent population genetics studies provide further support for the functional relevance of these elements through evidence of sequence constraint [65]. Therefore, the enrichment of short uORFs in the Primary set is likely driven by biological factors, such as the overall higher level of translation for uORFs compared to lncRNA-ORFs, dORFs, and others [26], rather than false positives from the original ORF callers or artifacts from experimental protocols, as the included datasets are not biased to incorporate cells treated with antibiotics (e.g. homoharringtonine, lactimidomycin) that stall ribosomes in the 5′ UTR [66, 67]. Another key feature in this catalog is the inclusion of various isoforms of a given ncORF, such as different splicing isoforms or alternative start or stop positions. We included these for three main reasons. First, they likely reflect genuine transcriptional and translational complexity rather than technical artifacts. Second, comprehensive annotation supports efforts to characterize translation and enables tracking of isoforms that may be differentially detected across studies, because different RNA isoforms can expose alternatively translated ncORFs whose expression varies across cell types, tissues, or conditions. Third, given the current limitations in assigning function, excluding isoforms prematurely may overlook biologically relevant candidates not captured in the Primary set.

Nevertheless, we acknowledge the limitations of this work. First, our usage of published ncORF calls from a limited number of publications may have a bias toward ncORFs identified in specific samples, by specific research groups, or by certain Ribo-Seq analysis tools/protocols. Equally, the use of two large ribosome profiling resources used in the creation of our translational signatures may result in the exclusion of known (or real) ncORFs because of the relative under-representation of the cell types or tissues in which they are found. Second, trusting the ncORF calls made by other publications necessitates accepting decisions made about pre-processing and post-processing steps of the data, which may differ between research efforts. Notably, recent machine learning approaches employ no preprocessing steps for the data [68] or use sequence information to improve the prediction of Ribo-seq signal [69–71]. Third, scoring metrics such as PIF and Uniformity are biased toward certain ncORFs, particularly small ones (as noted above) and biased against those overlapping with other translated reading frames or CDSs. In overlapping ncORFs, periodic ribosome footprint signals become mixed, making their independent evaluation difficult and leading to an underestimation of their presence [22]. Fourth, start codon determination and assigning the correct RNA isoform to each ncORF remain challenging. Fifth, as GENCODE does not provide annotation for circRNAs, ncORFs originating from them are not incorporated. Last, here, we refer to these translation regions as ncORFs, noting that this is not a codified term in our databases and we anticipate a new ontology system will be developed in due course. For all of these reasons, we regard reference annotation to be an ongoing and iterative process and hope to be able to better address these potential concerns in future catalog iterations as more Ribo-seq datasets are incorporated and methods for ncORF detection improve.

Although the Primary set is unlikely to represent a complete survey of ncORFs with biological relevance, we expect the filtering approach taken here increases the rate of accurate ncORF detection and provides a subset of ncORFs with the highest utility for the life sciences and medical communities. Moreover, our resource provides a comprehensive overview of Ribo-seq evidence for all ncORFs included in both the Primary and Comprehensive sets. Researchers can customize their analyses by selecting their own thresholds for PIF, Uniformity, and Drop-off scores or by filtering based on additional metadata—such as expression levels or ORF length (Supplementary Table S2 and  S4). This flexibility enables optimization for various downstream applications, including mass spectrometry, immunopeptidomics [13], evolutionary analyses [11, 13], testing for degradation signals [72, 73], interaction network mapping [74], and other strategies aimed at identifying functional candidates aligned with specific research goals.

In summary, we present here an expanded catalog of ncORFs aligned with the GENCODE v45 resource and support a Primary set of 10 127 ncORFs that have high-quality evidence of their translation. We believe that this work will be widely applicable to future biomedical research on ncORFs in diverse settings, including human health and disease.

## Supplementary Material

gkag234_Supplemental_Files

## Data Availability

The data underlying this article are available in https://www.gencodegenes.org/pages/riboseq_orfs/ as well as in the online supplementary material. Human body-map raw data can be downloaded from GEO superseries GEO: GSE182377. Ribocrypt data can be accessed at https://ribocrypt.org/. The ncORF lists from individual lists can be found in their respective publications https://doi.org/10.7554/eLife.08890, https://doi.org/10.1038/nmeth.3688, https://doi.org/10.7554/eLife.13328, https://doi.org/10.1016/j.cell.2019.05.010, https://doi.org/10.1038/s41589-019-0425-0, https://doi.org/10.1126/science.aay0262, https://doi.org/10.7554/eLife.58659.

## Notes

Present address: Institute of Molecular Systems Biology ETH Zurich, Zurich 8093, Switzerland
