# Supplementary material for: An expanded reference catalog of translated open reading frames for biomedical research
Source: Nucleic Acids Res. 2026 Mar 24;54(6):gkag234. doi: 10.1093/nar/gkag234 (PMC13010147; doi:10.1093/nar/gkag234)
Supplement: gkag234_Supplemental_Files [file gkag234_supplemental_files.zip › GENCODEv45_supplementary_figures.docx]

**
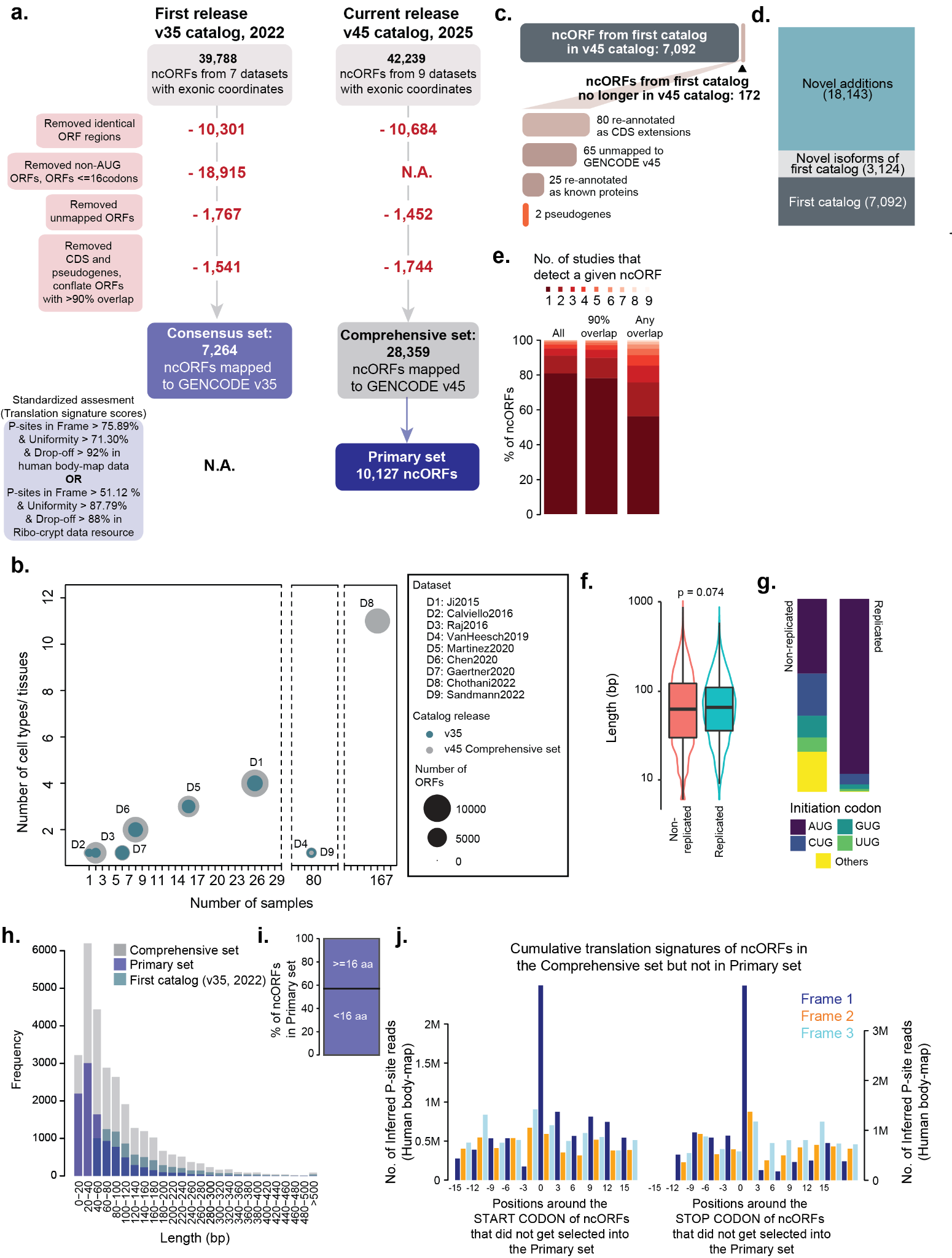
Supplementary Figure 1.** **a)** A schematic of the filtering steps for the previously published catalog in 2022 (v35) and the current v45 catalog. Numbers in red denote ncORFs that were removed. **b)** A bubble plot showing number of cell types or tissues vs number of samples sequenced with circle size denoting the number of ncORFs identified in a given study. Light blue: NcORFs retained in v35 catalog. Gray: NcORFs retained in v45. **c)** Number of ncORFs from the v35 catalog that were retained/not retained in the updated v45 catalog. Re-annotated to CDS includes cases that overlapped annotated proteins with missing 5’ or 3’ ends, or newly annotated extensions which have now been updated on GENCODE. **d)** Stacked barplot showing number of ncORFs included in the v45 catalog that were also included in the first catalog (v35 catalog, n=7,092), new additions that are isoforms of ncORFs included in the first catalog (n=3,124), and novel additions to this v45 catalog (n=18,143). **e)** Stacked bar plot illustrating the overlap of ncORF sequences identified across nine studies. The analysis includes all individual ncORFs (left), ncORFs clustered based on 90% sequence overlap (middle), and ncORFs grouped by any degree of overlap (right). **f)** Box plot showing the length differences between replicated and non-replicated ncORFs. Wilcoxon test, p-value = 0.074. **g)** Stacked bar plot illustrating the proportion of initiation codons in replicated and non-replicated ncORFs. **h)** Barplot showing length distribution of ncORFs. Gray: v45 catalog, Comprehensive set, Dark blue: v45 catalog, Primary set, Light blue: first catalog (v35 catalog, 2022). **i)** Barplot showing percentage of ncORFs up to 16 codons in length, threshold used in the first catalog (GENCODE v35). **j)** P-site profiles using human body-map dataset around the START and STOP codon of ncORFs in the Comprehensive set that did not get selected into the *Primary* set. Dark blue: P-sites in Frame 1, Orange: P-sites in Frame 2, Light blue: P-sites in Frame 3.

**
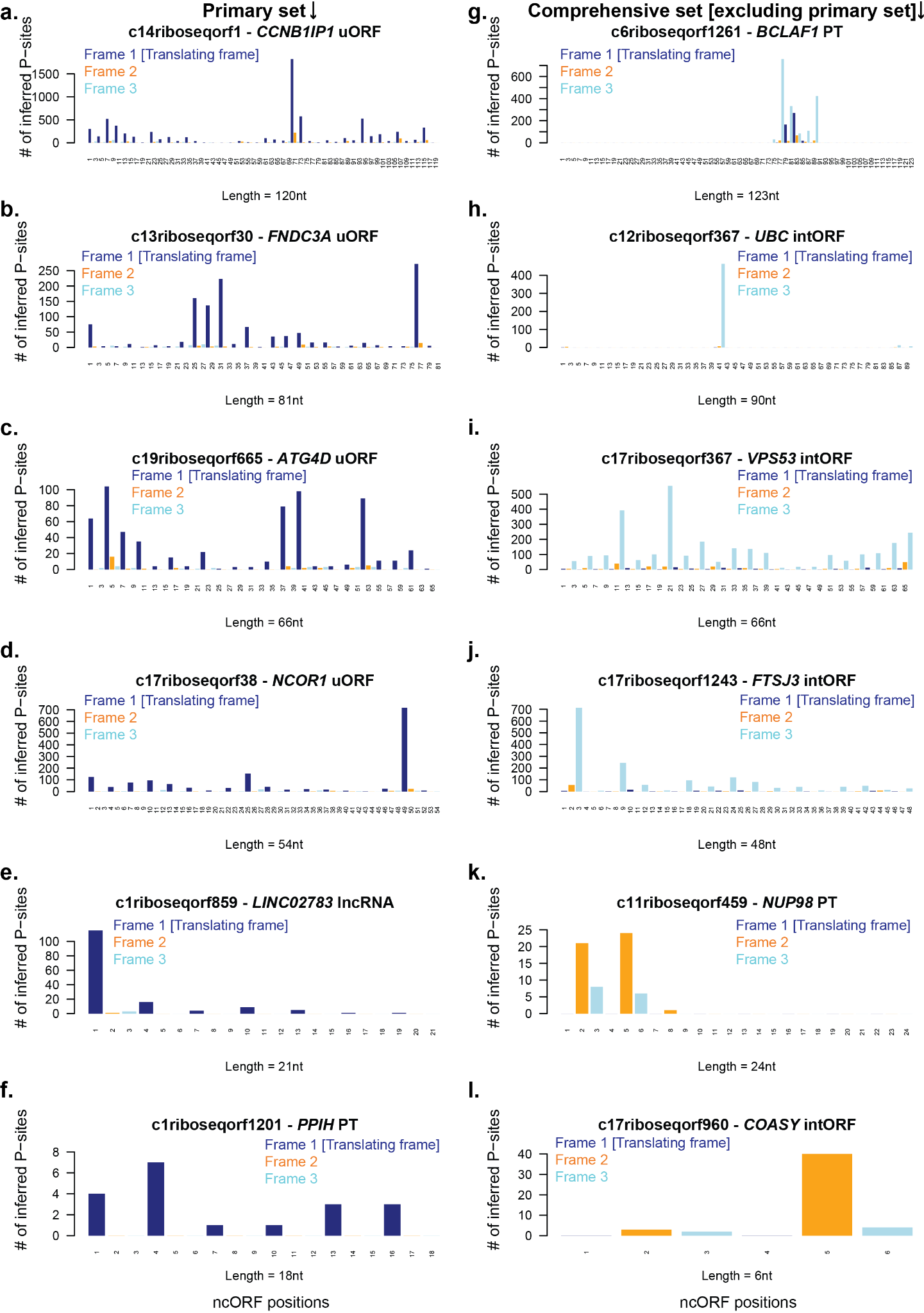
Supplementary Figure 2:** Individual examples of high-scoring and low-scoring ncORFs. **a-f.** Barplots showing number of P-sites throughout the length of high-scoring ORFs. **g-l.** Barplots showing number of P-sites throughout the length of low-scoring ORFs. Dark blue: Translating frame/Frame 1, Orange: Frame 2, Light blue: Frame 3.

**
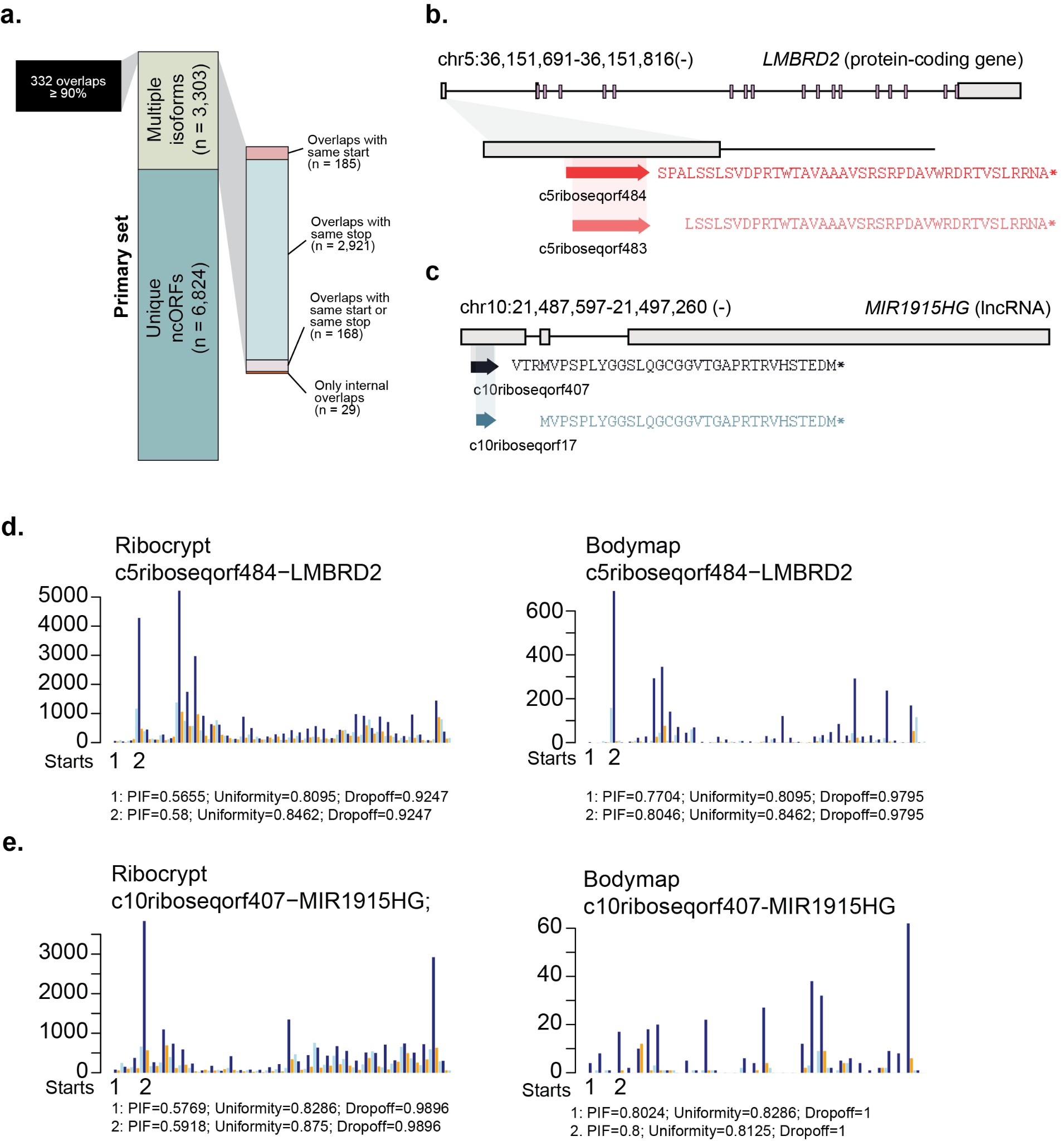
**

**Supplementary Figure 3. a.** Proportion and number of ncORFs in the Primary set, categorized based on the presence or absence of additional ncORF isoforms. For the set of 3,303 ncORFs associated with multiple isoforms, the number of overlapping isoforms (≥90% codon overlap with the shortest isoform) is indicated, as this threshold was used in the first catalog to collapse ncORFs. Additionally, all 3,303 ncORFs with multiple isoforms are further stratified by the type of overlap with other ncORFs. b. Two overlapping uORF variants located in the LMBRD2 gene have near-cognate initiation codons. **c.** A translated region located in the *MIR1915HG* lncRNA has two different predicted initiation codons utilizing either an AUG initiation codon or a near-cognate initiation codon. **d-e**. P-site profiles for ncORFs in LMBRD2 (d) and MIR1915HG (e) using human body-map and RiboCrypt data with the different initiation site marked as “starts” 1 and 2.

**
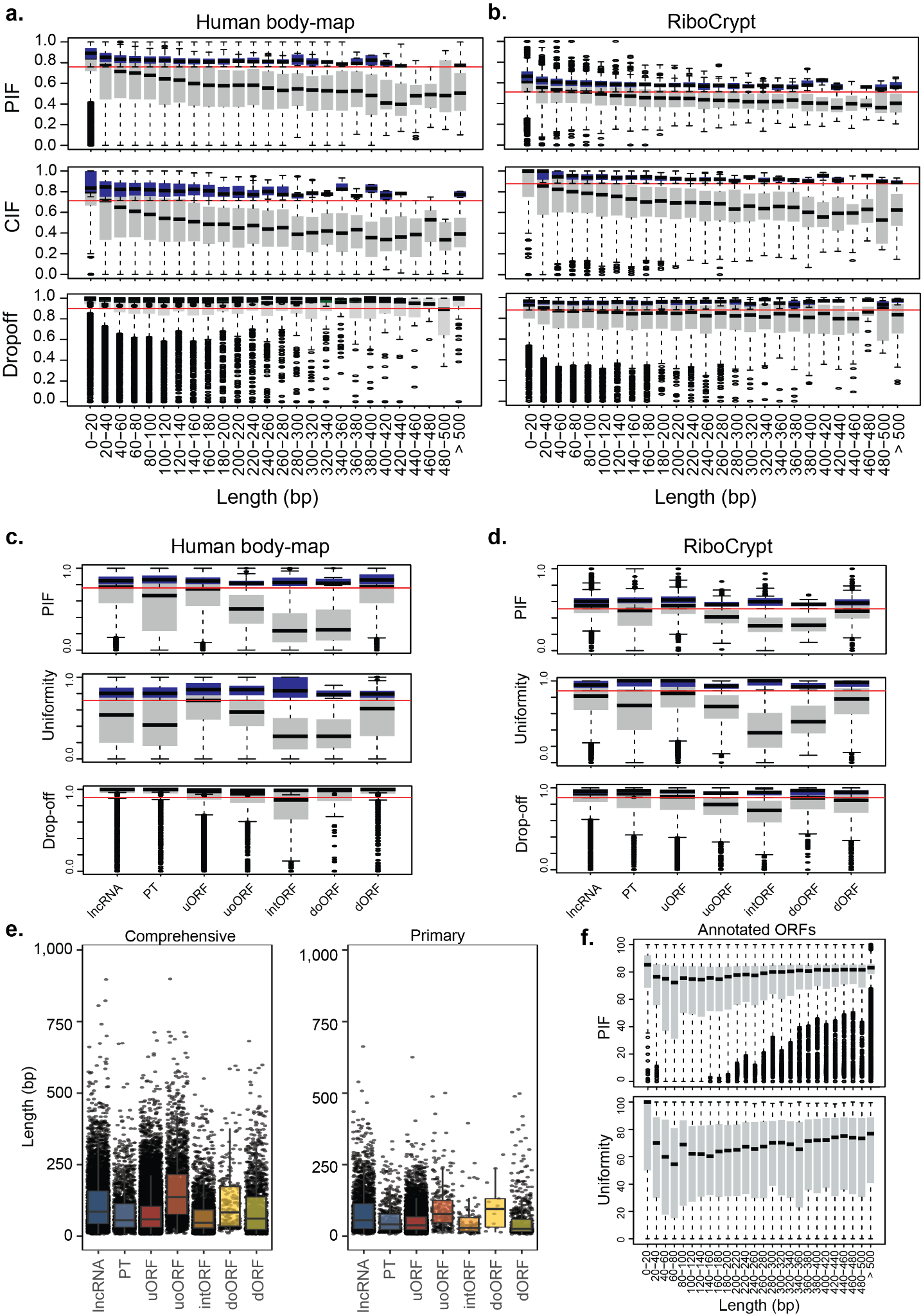
Supplementary Fig. 4. a-b.** Boxplot showing translation signature score (PIF, Uniformity and Dropoff) distribution across ORF length bins (in base pairs) in human body-map (a) and Ribocrypt data (b). **c-d.** Boxplot showing translation signature score (PIF, Uniformity, Dropoff) distribution across ORF types in human body-map (c) and RiboCrypt data (d). **e.** Boxplot showing length distribution of ncORFs across different ncORF types in the Comprehensive set and Primary set. **f.** Boxplot showing PIF and Uniformity score distribution across length bins of annotated ORFs.

**
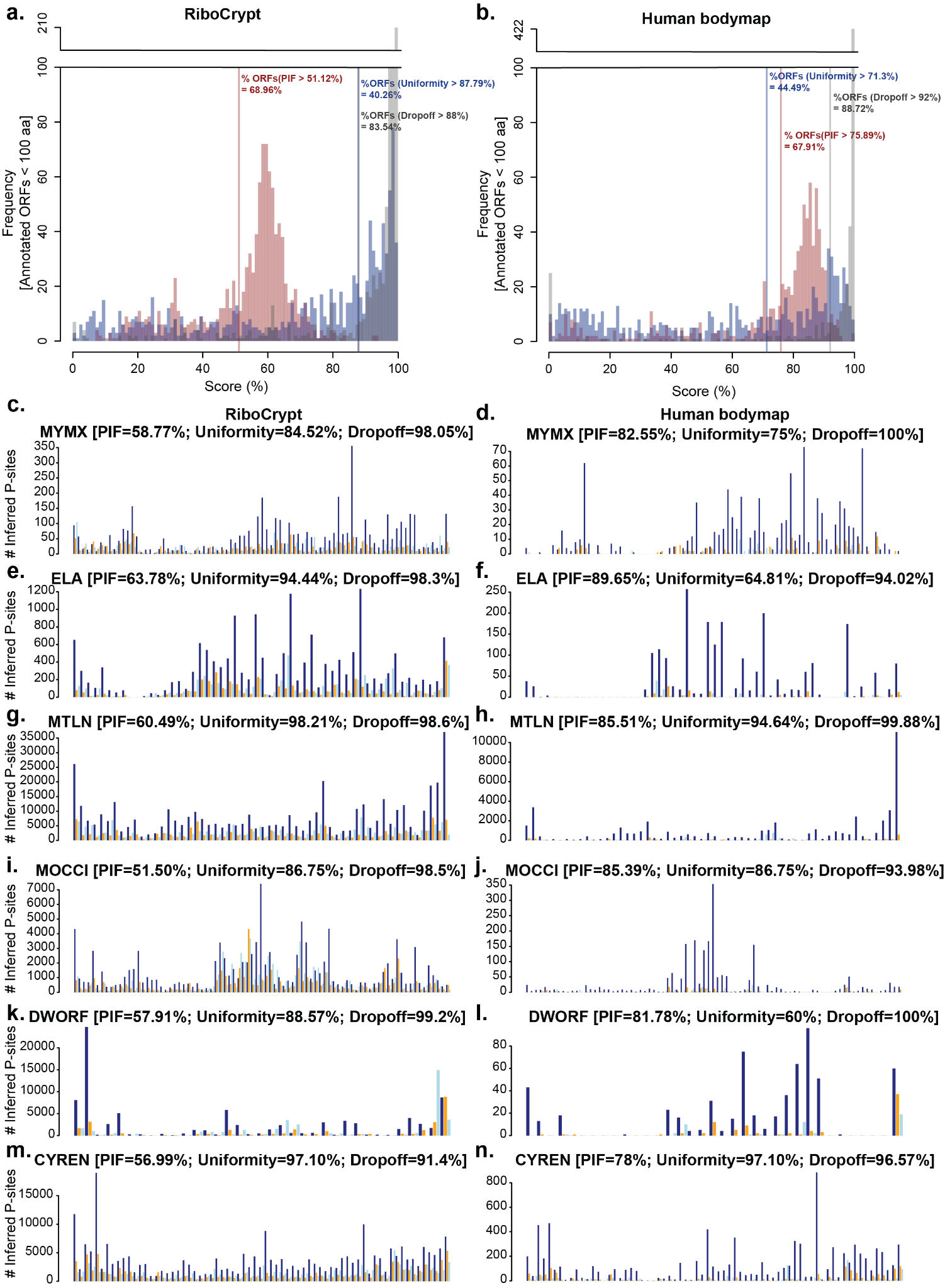
Supplementary Figure 5: Translation signature scores for annotated Ensembl ORFs less than 100 aa and known microproteins. a-b.** PIF, Uniformity and Drop-off score distributions for annotated ORFs less than 100aa using Ribocrypt **(a)**, and Human body-map dataset **(b)**. **c-n.** P-site profiles for six known characterized microproteins encoded by ncORFs including Myomixer (MYMX, **c-d**), Elabela (ELA, **e-f**), Mitoregulin (MTLN, **g-h**), MOCCI (**i-j**), DWORF (**k-l**) and CYREN (**m-n**), in RiboCrypt and human body-map respectively.

**Supplementary Tables**

Filename: Supplementary_Tables_NAR_20260130.xlsx

**Supplementary Files**

Supplementary File 1: Three-nucleotide periodicity around the start- and stop- codon of the ncORFs in Primary set and remaining comprehensive set across various ORF types. Page 1: Human body map data, Page2: RiboCrypt data
