# Supplementary material for: An expanded reference catalog of translated open reading frames for biomedical research
Source: Nucleic Acids Res. 2026 Mar 24;54(6):gkag234. doi: 10.1093/nar/gkag234 (PMC13010147; doi:10.1093/nar/gkag234)
Supplement: gkag234_Supplemental_Files [file gkag234_supplemental_files.zip › Supplementary_file_1.pdf]

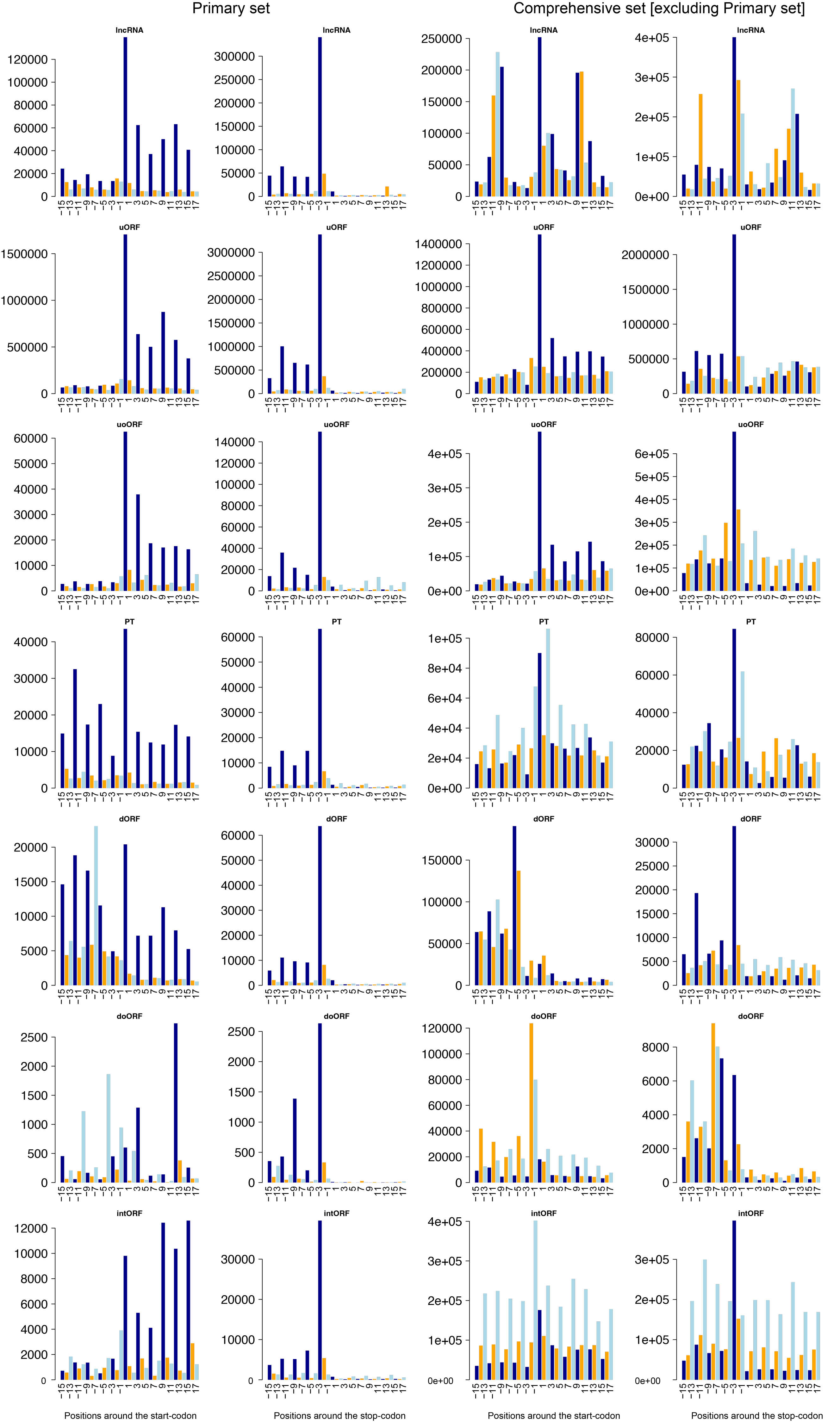

Primary set
Comprehensive set [excluding Primary set]
lncRNA
uORF
uoORF
PT
dORF
doORF
intORF
Positions around the start-codon
Positions around the stop-codon
Positions around the start-codon
Positions around the stop-codon

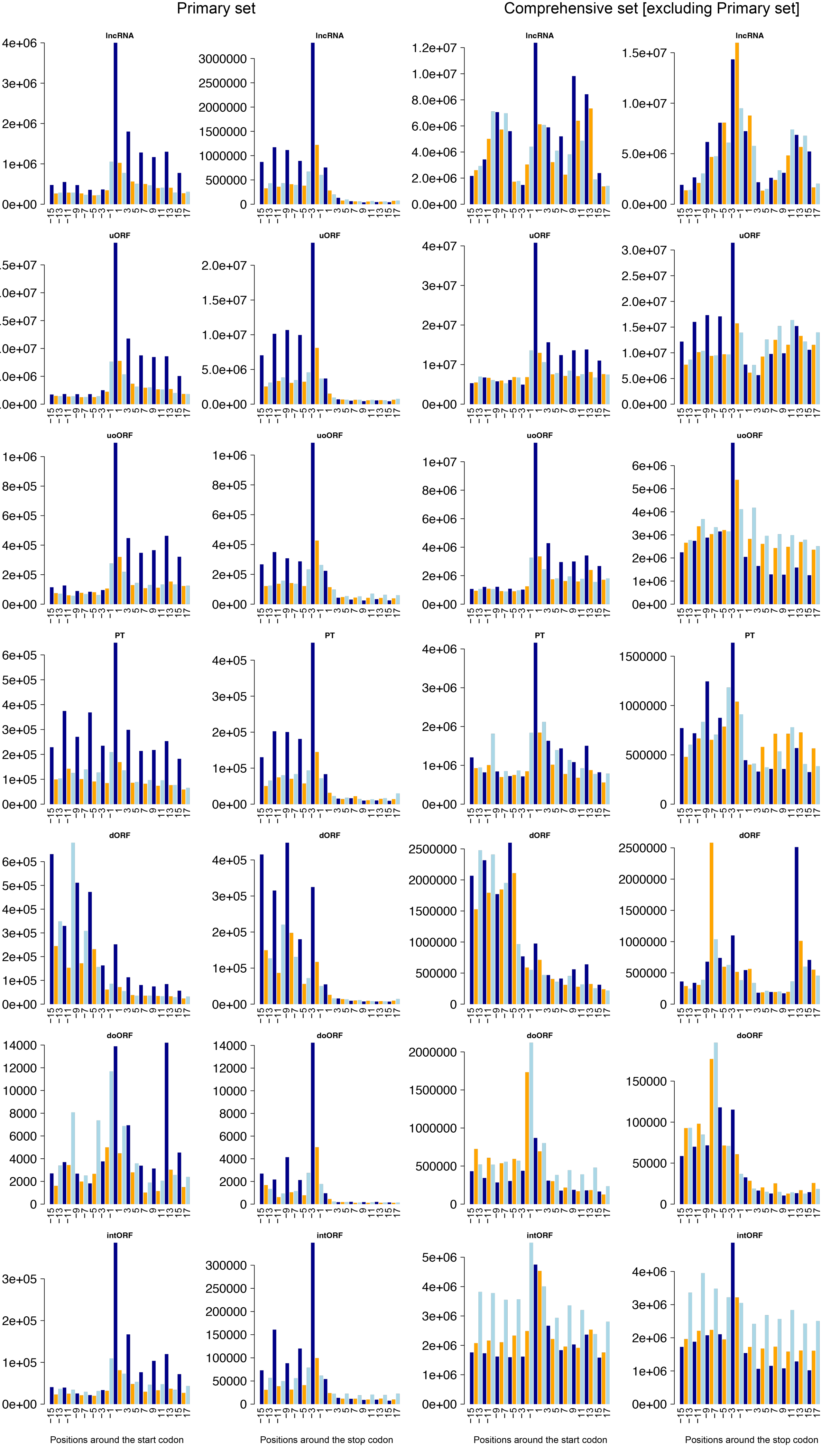
Primary set
Comprehensive set [excluding Primary set]
lncRNA
uORF
uoORF
PT
dORF
doORF
intORF
Positions around the start codon
Positions around the stop codon
Positions around the start codon
Positions around the stop codon
